# New species without dead bodies: a case for photo-based descriptions, illustrated by a striking new species of *Marleyimyia* Hesse (Diptera, Bombyliidae) from South Africa

**DOI:** 10.3897/zookeys.525.6143

**Published:** 2015-10-05

**Authors:** Stephen A. Marshall, Neal L. Evenhuis

**Affiliations:** 1 Department of Environmental Biology, University of Guelph, Guelph, Ontario N1G 2W1, Canada; 2 J. Linsley Gressitt Center for Entomological Research, Bernice Pauahi Bishop Museum, Kaiwi‘ula Campus, 1525 Bernice Street, Honolulu, Hawaii 96817-2704, USA

**Keywords:** South Africa, bee fly, mimicry, *Xylocopa*, type specimens, photography, taxonomy

## Abstract

A new bombyliid species *Marleyimyia xylocopae* Marshall & Evenhuis, **sp. n.**, an apparent mimic of the carpenter bee *Xylocopa flavicollis* (De Geer), is described from South Africa on the basis of photographs only. The pros and cons of species descriptions in the absence of preserved type specimens are discussed.

“*Collecting specimens is no longer required to describe a species or to document its rediscovery.*”—Minteer et al. (2014: 260)

“*Describing a new species without depositing a holotype when a specimen can be preserved borders on taxonomic malpractice.*”—Krell and Wheeler (2014: 815)

## Introduction

A recent paper in *Science* by Minteer et al. (2014) ignited a controversy by suggesting that specimens are no longer the ‘gold standard’ for species description, and that alternative methods such as high-resolution photography should be considered instead. The quotes above, from the Minteer et al. (2014) paper and a response letter by Krell and Wheeler (2014), give some indication of the apparent polarization of the community on this issue. We take the opportunity here to contend that the apparently antipodal positions summarized in those quotes are both correct, and provide a description of a new species based only on photographs to illustrate and confirm our points.

First of all, let us reiterate the obvious reasons that collecting specimens is highly desirable, and briefly consider and reject arguments against collecting. Specimens are indeed the ‘gold standard’ for species descriptions. Not only do they allow for consideration of a full suite of characters including internal morphology, microscopic and genetic characters, they preserve data for future access with future technologies and future questions. Specimen collections are our greatest treasure trove of biodiversity information and continued collection development must remain a priority. Arguments against specimen collections usually pivot on the potential impact of collecting on fragile populations. Such arguments are weak, since there are vanishingly few examples of scientific collecting having a detrimental effect at the population or species levels and there are very few circumstances under which the removal of a few individuals from a population might seriously harm the ultimate survival of the species. Most, by orders of magnitude, of the animal species awaiting description are invertebrates, and it is especially difficult to make a case against invertebrate collecting on the basis of conservation biology. As invertebrate taxonomists, we therefore agree with the letter of Krell’s (2014) statement that “*Describing a new species without depositing a holotype when a specimen can be preserved borders on taxonomic malpractice*” (p. 815), but at issue here is the caveat of when a specimen can be preserved. We do not accept opposition to killing and preserving invertebrate specimens on moral or conservation biology grounds, but there are circumstances under which a type cannot be preserved. Furthermore, there is no doubt that collection of potential type specimens will become more and more difficult as restrictions on collecting and transporting specimens continue to increase. These difficulties, along with the rapidly increasing numbers of skilled ‘digital collectors’ who are building collections of images instead of specimens, will inevitably force the biodiversity community to adapt to growing numbers of new taxa recognized without benefit of dead, preserved type specimens. Fortunately, as Minteer et al. (2014) put it, “*collecting specimens is no longer required to describe a species*.... (p. 260)”. Collecting specimens is highly desirable, but it is indeed no longer required.

As explained by Wakeham-Dawson et al. (2002) and Polaszek et al. (2005), although Article 16.4 of the ICZN Code (ICZN 1999) requires all holotypes that are “extant” to be deposited in a collection, Article 73.1.4 allows for the description of new taxa without preserving a collected specimen by the following statement: “*Designation of an illustration of a single specimen as a holotype is to be treated as designation of the specimen illustrated; the fact that the specimen no longer exists or can be traced does not of itself invalidate the designation*”. Additionally, we interpret the wording of Article 16.4 to allow for description of a new species on the basis of a lost or escaped holotype, where the term “extant” means a physically “existing” specimen. Thus a lost, escaped, or purposefully released specimen is not “extant”.

Even in the absence of a collected type specimen, current technologies such as high-resolution photography can often provide enough information for a proper description resulting in a readily recognizable and unequivocally distinct newly named species, and in some cases can provide more information (such as colour, soft parts, delicate structures, posture, behaviour) than could be extracted from a preserved specimen. The few previous descriptions of extant new species without a type (or part thereof) have for the most part been restricted to large vertebrates, for example primate species known from only small populations (Jones et al. 2005, Li et al. 2015, Mendes Pontes et al. 2006). We provide the first example of a new insect species described and named solely on the basis of field photographs of the type specimen.

In the example provided here, a highly distinctive fly species belonging to an extremely rare genus (only three other known specimens of two species) was photographed on two occasions and then collected in the field, but the captured individual escaped before it could be preserved as a traditional dead type specimen. Our description, based on photographs of two different living flies, is complete and adequate to identify this species and adds an interesting and easily recognized species to the literature. It not only increases our knowledge of the biodiversity of the area in which it was collected and of the genus in which it is placed but, as we explain below, also provides some interesting ecological and biological information.

The situation leading to this approach is a simple one to understand, since it pivots on an accidentally lost type specimen that might not be collected again due to its rarity. Although this description is by definition singular because it is the first of its kind (at least for Diptera), we predict that a growth in descriptions without physical type specimens is inevitable, and that this growth will result not as much from accidental loss of specimens as from increasing restrictions on collecting. Every taxonomist has been in the position of completing a revision that needs to be rounded out with species from places from which specimens are difficult or impossible to obtain, often because of laws preventing collection or export of specimens. For many of these required taxa, the solution to this problem is for the taxonomist to instead “collect” digital images that, in the case of new species, can represent type specimens. We are not arguing that this practice is generally desirable, only that it is inevitable and increasingly practical when diagnostic characters are distinct and discernable through photographs.

Another trend pushing us inexorably to a wider acceptance of species descriptions without physical type specimens is the rapid growth of extensive, high quality digital image collections dissociated from collections of physical specimens (Marshall, in press). As these image collections become curated just as dead specimens are curated today, these digital “specimens” will find their way into the work of practicing taxonomists, and they will need names. At a time when we need more than ever to identify the biodiversity of this planet before it disappears, it is unrealistic to think that distinct and diagnosable new taxa known only from good photographs and appropriate associated metadata should be organized and referred to only as “undescribed species #nnn”, when they can and should be organized and named using the existing rules of nomenclature.

In recognizing the need to name species without dead type specimens we are not arguing for a loosening of taxonomic standards. In fact, we expect that descriptions unsupported by existing physical type specimens will be subject to especially critical scrutiny by skeptical editors and responsible reviewers. We expect that such descriptions that do not render new species unequivocally recognizable will be rejected, just as they should be if they were based on dead type specimens. Once published, digital representations of type specimens will be much more widely available for use and scrutiny than physical type specimens archived in distant museums.

Dubois and Nemésio (2007) argue specifically against the use of photographs as surrogates for type specimens, suggesting that digital photographs can be faked or misinterpreted. This is, of course true, but one could also alter or misinterpret a type specimen. We see no merit in impeding the documentation of biodiversity on the baseless assumption that there will be more errors, incompetency or dishonesty in descriptions based on photographs than currently exists in descriptions based on specimens or parts thereof.

## Material and methods

The species described below was photographed in nature using a Nikon D800 with a 105 macro lens and a hand-held flash. The holotype specimen was not captured, so the image presented serves as representation of the holotype. Morphological terminology follows Greathead and Evenhuis (2001) and wing venation follows Saigusa (2006).

## Taxonomy

### Genus Marleyimyia

Taxon classificationAnimaliaDipteraBombyliidae

Hesse

Marleyimyia Hesse, 1956: 521. Type species: *Marleyimyia natalensis* Hesse, 1956, by original designation.

#### Remarks.

*Marleyimyia* Hesse, 1956 was originally described based on a single male specimen with vestigial mouthparts and bred from a log containing cossid larvae. The genus is currently known from only three specimens representing two described species from widely disjunct localities: *Marleyimyia goliath* (Oldroyd) from Peninsular Malaysia and *Marleyimyia natalensis* Hesse from southern Africa. In proposing his new genus, Hesse (1956) distinguished *Marleyimyia* from *Oestranthrax* Bezzi, 1921 by the larger body, the head wider than the thorax, and the differently shaped, although reduced, proboscis (pointed apically in *Marleyimyia* and short with a small fleshy labellum in *Oestranthrax*). Hesse distinguished *Marleyimyia* from *Villoestrus* Paramonov, 1931 by the same body and head features as above, but also the presence of a reduced proboscis (proboscis totally absent in *Villoestrus*). Oldroyd (1951) described his new Malaysian species as *Oestranthrax goliath* based on a single male and female bred from the pupa of a cossid moth and claimed it to be the largest in bulk of any bee fly he had seen. Bowden (1975) transferred *Oestranthrax goliath* to *Marleyimyia* and Bowden (1978) echoed Oldroyd’s (1951) presumption that the species in the genus had the appearance of a crepuscular or nocturnal habit. If this nocturnal habit is proven to be true, then the new species described below differs in having been seen during the day (photographed at two separate localities), but it has the same unusual antennal shape as found in the male and female of *Marleyimyia goliath* (a similar lanceolate shape but shorter and stouter is found only in one other bombyliid species, the Nearctic *Oestranthrax farinosus* Johnson & Maughan, and only in females of that species). The antennal shape in the new species described here is not found in the male holotype of the type species, *Marleyimyia natalensis*, but it is found in the female of the undescribed species of *Marleyimyia* from Nigeria mentioned by Bowden (1978) and may be a female-specific character for species of *Marleyimyia*.

### Marleyimyia xylocopae

Taxon classificationAnimaliaDipteraBombyliidae

Marshall & Evenhuis sp. n.

http://zoobank.org/02D005F8-59D4-4FD7-B0D2-FAA8BC017BC8

Figs 1 , 2 , 3 , 4

#### Type locality.

REPUBLIC OF SOUTH AFRICA: KwaZulu Natal: Ndumo Nature Preserve, Ndumo Campground, 26°54'31.07"S; 32°18'57.85"E.

#### Type specimen.

Holotype female from SOUTH AFRICA: KwaZulu Natal: Ndumo Nature Preserve, Ndumo Campground, 26°54'31.07"S; 32°18'57.85"E, 74.0 m elev., 1 Dec 2014, S.A. Marshall. Holotype represented in photograph No. 7007 (Fig. 1); other photos taken: Nos. 7002, 7003, 7004, 7005, 7006, 7008. Paratype female represented in photograph No. 7015. photographed at the following locality: SOUTH AFRICA: KwaZulu Natal: Ndumo Nature Preserve, Red Cliffs, 26°51'21.9"S; 32°12'26.3"E, 35.0 m elev., 27 November 2014, S.A. Marshall. Other photos taken: Nos. 7009, 7010, 7012, 7013, 7014, 7016, 7017. All photographs are archived with Morphobank (project P2277 : http://morphobank.org/permalink/?P2277, images M397297–M397315).

**Figure 1.**
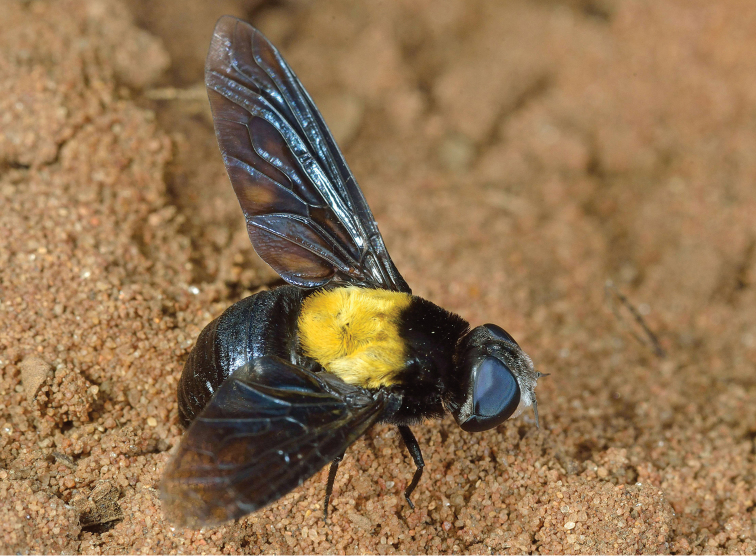
Habitus of female *Marleyimyia xylocopae* Marshall & Evenhuis, sp. n. from Ndumo Game Preserve, South Africa, 1 December 2014. Fig. 1 derives from Photo no. 7007 (Campground site) and was adjusted for brightness and contrast. Photo: Steve Marshall.

#### Diagnosis.

Separated from its congeners by the all black infuscated wing (hyaline in *Marleyimyia goliath* and *Marleyimyia natalensis*), and the mesonotal pattern of black hairs anteriorly and yellow hairs posteriorly (entirely black-haired in *Marleyimyia goliath* and predominantly yellowish brown-haired in *Marleyimyia natalensis*).

#### Description.

**Figure 2.**
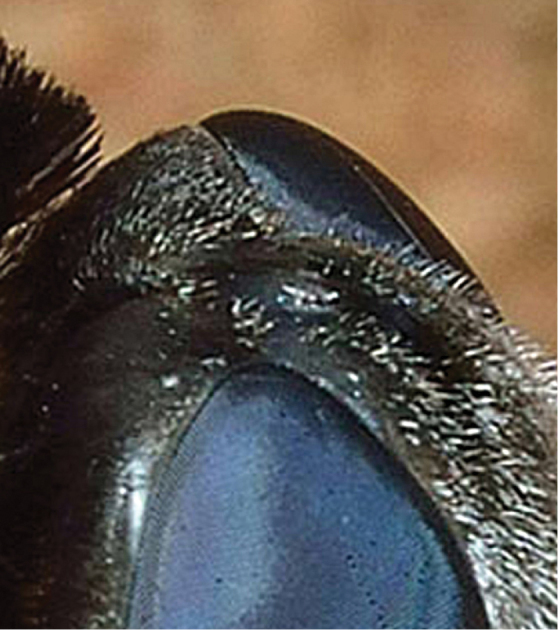
*Marleyimyia xylocopae* Marshall & Evenhuis, sp. n., detail of head. Photo: Steve Marshall.

**Figure 3.**
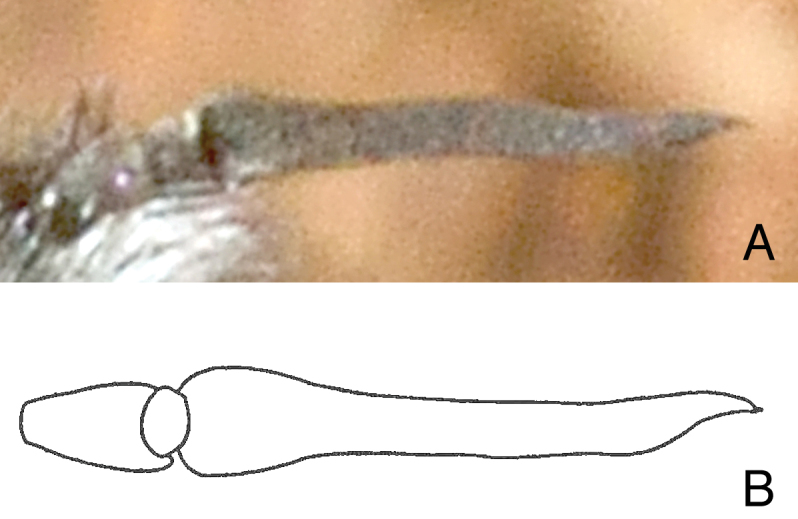
Right antenna of *Marleyimyia* species. **A** *Marleyimyia xylocopae* Marshall & Evenhuis, sp. n. **B** *Marleyimyia goliath* (Oldroyd) [from Oldroyd 1951, fig. 5]. Fig. 3A derives from Photo no. 7015 (Red Cliffs site), was rotated slightly to match the illustration (Fig. 3B), and was adjusted for brightness and contrast. Photo: Steve Marshall.

Female. Body: ca. 18–20 mm in length (extrapolated from comparison of grass blade width of *Eremochroa* (centipede grass) in a larger habitus photograph [No. 7009]). *Head* (Fig. 2). As wide or wider than thorax, shining black in ground colour with some bluish highlights, clothed with silvery white hairs and tomentum. Frons width ca. 1/3 of head width at occiput; ocellar triangle ellipsoid, lateral ocelli slightly larger than anterior ocellus; occiput with short silvery white hairs dorsally, silvery white tomentum along posterior eye margin, tomentum densest at medial eye indentation and on postgena. Eye dark bluish black, indented medially on posterior margin, with bisecting line length ca. half eye width. Frons short silvery white pilose and tomentose, bare medially below ocellar triangle, pile longest and densest at level of antennae. Face receding with dense silvery white hairs, oral margin narrowly brownish orange near eye margin. Antenna (Fig. 3A) cinereous; scape subcylindrical with admixed black and white hairs dorsally and laterally; pedicel subellipsoid, wider than long, bare; flagellomere long, ca. 4 × length of scape, linear-lanceolate, bare, slightly bulging basally, slightly tapering to apex; apical style minute.

*Thorax*. Mesonotum and pleura shining black in ground color (scutellum ground color obscured); mesonotum with dense short “clipped-looking” black pile anteriorly to level of wing base, yellow pile from wing base to posterior edge of mesonotal disc including postalar calli, long, shaggy laterally, short and “clipped-looking” on disc; scutellum densely shaggy yellow pilose; pleura thickly black haired, those hairs on anepisternum with dark brownish sheen. (Halter and pleural area under wing obscured in photos).

*Legs*. (Hind femur obscured in photographs). Fore and mid legs (and hind legs beyond femur) black with a shiny greasy appearance, some bluish highlights on femora and tarsi. Fore and mid femora short, stout, with long black hairs ventrally, longest basally, tapering to shorter apical hairs; tibiae shorter than femora, with short black spicules.

**Figure 4.**
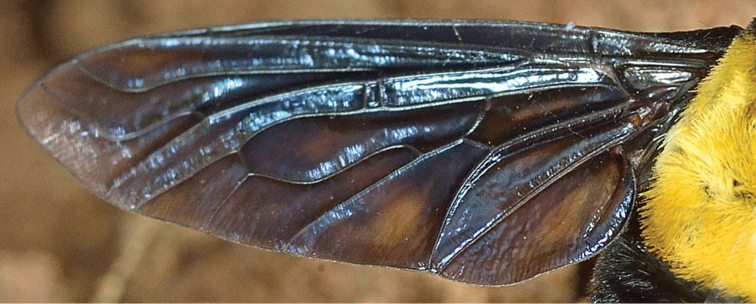
*Marleyimyia xylocopae* Marshall & Evenhuis, sp. n., left wing. Fig. 4 derives from Photo no. 7007 (Campground site) and was adjusted for brightness and contrast. Photo: Steve Marshall.

*Wing* (Fig. 4). Infuscated dark brownish black throughout except brownish infuscation in center of anal lobe and cell fourth posterior cell and subhyaline apex of wing, veins black; crossvein r-m just proximal to middle of cell dm (with anomalous second crossvein in left wing); veins R_2+3_ and R_4_ sinuous, subparallel to wing margin; origin or R_2+3_ just before r-m crossvein; first posterior cell open in wing margin; crossvein dm-m S-shaped, origin on vein M_4_ at basal one-fourth; crossvein m-m slightly wider than r-m; cell cua narrowly open in wing margin; anal lobe well developed; alula small.

*Abdomen.* Broad, ovular in shape, shining black in ground color with bluish highlights (sternites not visible); tergite II and III with admixed short silvery white hair and tomentum dorsolaterally and sparse silvery tomentum with bluish highlights dorsomedially; tergites IV–VII with adpressed black tomentum and sparse silvery white tomentum dorsomedially. *Genitalia*. Not dissected.

#### Remarks.

**Figure 5.**
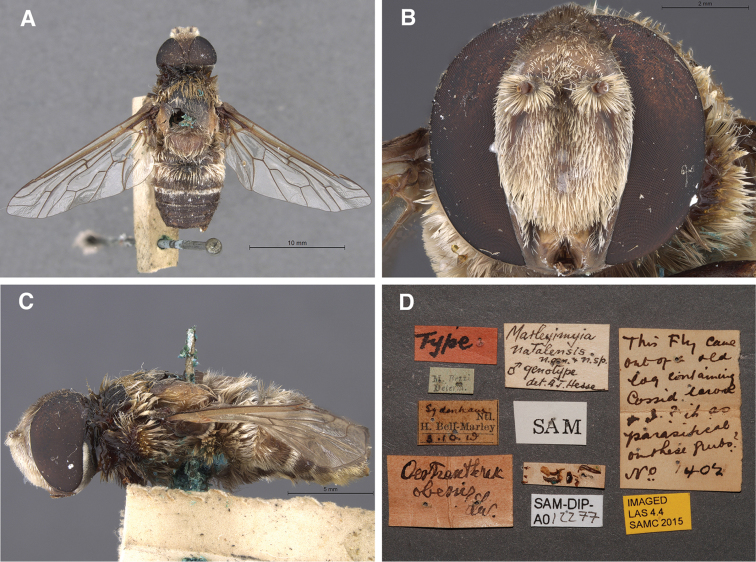
*Marleyimyia natalensis* Hesse, male holotype. **A** habitus, dorsal view **B** Head, frontal view **C** habitus, lateral view **D** labels. Photos: Simon Van Noort, Iziko South African Museum.

Two different specimens were photographed (one at each locality indicated above). That they are different is evidenced by the rubbed frons in the Red Cliffs paratype (photos taken on 27 November) and that the photos taken later at the Campground site (on 1 December) were of a specimen without a rubbed frons. This new species shares its unusual large body, wing shape, wing venation, and antennal flagellomere shape with *Marleyimyia goliath*, which occurs in Peninsular Malaysia. These characters differ from the smaller and more slender type species, *Marleyimyia natalensis* (Fig. 5), from Sydenham, near Durban, South Africa (see map in Fig. 7; http://www.simplemappr.net/map/4577). Bowden (1978) mentioned an undescribed species of *Marleyimyia* from Nigeria which, from photographs of the specimen in the BMNH, appears to be more similar to *Marleyimyia natalensis* in size and coloration than either *Marleyimyia goliath* or *Marleyimyia xylocopae* sp. n., but the Nigerian species has the same antennal shape as *Marleyimyia goliath* and *Marleyimyia xylocopae* sp. n. Because of the similarity of *Marleyimyia xylocopae* to characters shown in *Marleyimyia goliath*, we feel confident of its current generic placement. Further material of this genus should be secured in order to better assess the true generic limits.

## Discussion

**Figure 6.**
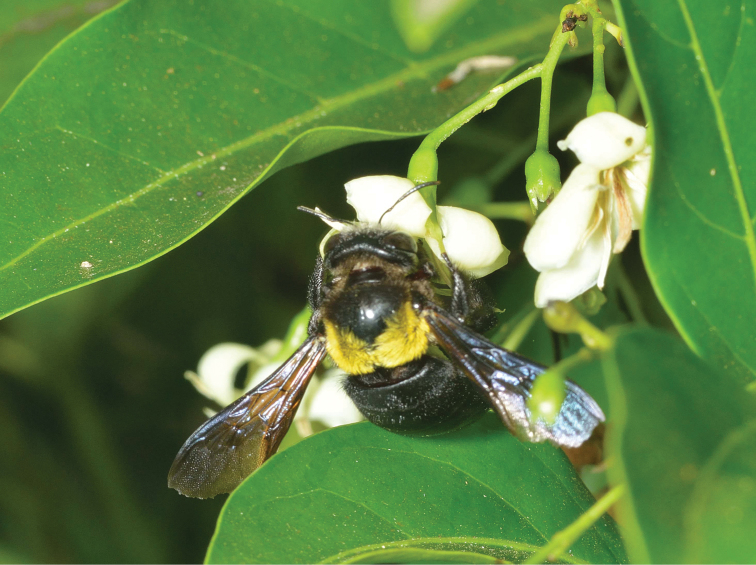
*Xylocopa flavicollis* (De Geer) from Ndumo Game Preserve, South Africa, 8 December 2014. Photo: Steve Marshall.

The striking yellow and black vestiture pattern on the thorax and abdomen, and the body shape, are unusual in bombyliids and show a remarkable similarity to xylocopid bees. The model for this possible case of Batesian mimicry appears to be *Xylocopa flavicollis* (De Geer), which was also photographed at around the same time in the Ndumo area (Fig. 6) and is fairly widespread throughout eastern and southeastern Africa. It is not known if *Marleyimyia xylocopae* is a parasite of the bee. Other bee fly parasites of *Xylocopa* are also unusually large-bodied and similarly shaped (e.g., *Xenox* Evenhuis and *Satyramoeba* Sack). Previous published records of African xylocopid biologies are few and the only recorded bee fly associate is in the genus *Anthrax* (Watmough 1974). More biological observations on xylocopid nesting behavior will hopefully lead to better knowledge of the full suite of their possible bee fly parasites.

**Figure 7.**
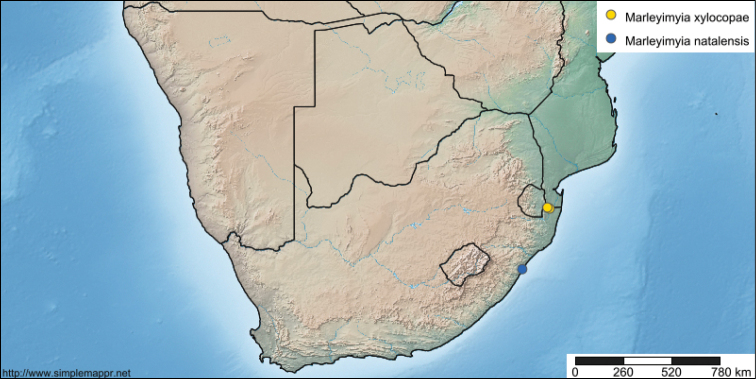
Map (http://www.simplemappr.net/map/4577) showing the known localities of *Marleyimyia natalensis* Hesse and *Marleyimyia xylocopae* sp. n., in Kwa-Zulu Natal, South Africa.

## Supplementary Material

XML Treatment for Marleyimyia

XML Treatment for Marleyimyia xylocopae
